# Cell cycle arrest combined with CDK1 inhibition suppresses genome-wide mutations by activating alternative DNA repair genes during genome editing

**DOI:** 10.1016/j.jbc.2024.107695

**Published:** 2024-08-17

**Authors:** Nozomi Fukuda, Keisuke Soga, Chie Taguchi, Jumpei Narushima, Kozue Sakata, Reiko Kato, Satoko Yoshiba, Norihito Shibata, Kazunari Kondo

**Affiliations:** 1 Division of Biochemistry, National Institute of Health Sciences, Kawasaki, Kanagawa, Japan; 2 Faculty of Food and Health Sciences, Showa Women’s University, Tokyo, Japan

**Keywords:** CRSIPR/Cas, genome-wide mutation, alternative DNA repair

## Abstract

Cells regularly repair numerous mutations. However, the effect of CRISPR/Cas9-induced dsDNA breaks on the repair processes of naturally occurring genome-wide mutations is unclear. In this study, we used TSCE5 cells with the heterozygous thymidine kinase genotype (*TK*^*+/−*^) to examine these effects. We strategically inserted the target sites for guide RNA (gRNA)/Cas9 and I-*Sce*I into the functional allele and designed the experiment such that deletions of > 81 bp or base substitutions within exon five disrupted the TK gene, resulting in a *TK*^−/−^ genotype. TSCE5 cells in the resting state exhibited 16 genome-wide mutations that affected cellular functions. After gRNA/Cas9 editing, these cells produced 859 mutations, including 67 high-impact variants that severely affected cellular functions under standard culture conditions. Mutation profile analysis indicated a significant accumulation of C to A substitutions, underscoring the widespread induction of characteristic mutations by gRNA/Cas9. In contrast, gRNA/Cas9-edited cells under conditions of S∼G2/M arrest and cyclin-dependent kinase 1 inhibition showed only five mutations. Transcriptomic analysis revealed the downregulation of DNA replication genes and upregulation of alternative DNA repair genes, such as zinc finger protein 384 (ZNF384) and dual specificity phosphatase, under S∼G2/M conditions. Additionally, activation of nucleotide and base excision repair gene, including O-6-methylguanine-DNA methyltransferase and xeroderma pigmentosum complementation group C, was observed. This study highlights the profound impact of CRISPR/Cas9 editing on genome-wide mutation processes and underscores the emergence of novel DNA repair pathways. Finally, our findings provide significant insights into the maintenance of genome integrity during genome editing.

The CRISPR/CRISPR-associated proteins (Cas) system has revolutionized genome engineering. This technology has been now extensively applied to the development of plant traits and gene therapies. The foundational CRISPR/Cas9 system from *Streptococcus pyogenes* comprises a guide RNA (gRNA) and the SpCas9 protein (1, 2). The gRNA identifies a 20 bp protospacer sequence that is complementary to the DNA target, whereas the Cas9 protein recognizes a three-base protospacer adjacent motif sequence on the opposite strand. This interaction forms an R-loop, allowing the gRNA–Cas9 complex to catalytically cleave the dsDNA at the specified site (3, 4, 5). Most double-strand breaks (DSBs) in cells are fixed by DNA repair mechanisms, such as nonhomologous end joining (NHEJ), microhomology-mediated end joining, and homology-directed repair. However, some DSBs can be restored by nucleotide deletions, insertions, or substitutions, which may result in gene knockouts.

Following the initial development of CRISPR/SpCas9, subsequent studies aimed to enhance SpCas9 fidelity, leading to the creation of high-specificity variants, such as SpCas9-HF (6) and HiFi-Cas9 (7). These variants have demonstrated precise editing capabilities in various cell lines (8). Another significant advancement was the discovery of the Cas12a (*Prevotella* and *Francisella* 1, Cpf1) ortholog, which exhibits a different protospacer adjacent motif recognition and cleavage pattern from those of SpCas9. Furthermore, Cpf1 generally produces precise edits with staggered ends (9, 10, 11, 12, 13).

Despite these efforts, SpCas9 remains the preferred tool for genome editing owing to its well-characterized effects on on-target and off-target sites. However, the off-target activity of CRISPR/Cas systems poses a significant concern, particularly for therapeutic applications, which have prompted extensive mitigating studies (14, 15, 16, 17, 18, 19). Although the high-fidelity Cas9 variants show improvements, they exhibit variable efficiencies and residual off-target activities, which could limit their clinical utility. Moreover, studies indicates that DNA repair outcomes are influenced by the protospacer sequence and are consistent across different cell lines, suggesting a nonrandom, context-dependent repair process (20). Deletion lengths range from a few to 20 bases (21, 22). The NHEJ pathway is the primary mechanism for DNA repair, which act immediately to inhibit large deletions and functions prominently even during the G2 phase when homologous recombination (HR) is active (23). A example of complex DNA breaks or chromatin rearrangement repair occurs if NHEJ cannot rapidly rejoin DNA ends, which initiates end resection and HR repair (24). Notably, larger deletions occur at low frequencies but are more pronounced in cells deficient in NHEJ pathway components, such as DNA ligase IV (LIG4), XRCC4, DNA-PKcs, and XLF. Conversely, deficiencies in Nijmegen breakage syndrome 1 (part of the MRE11–RAD50–Nijmegen breakage syndrome 1 complex) and DNA polymerase theta (POLQ) result in reduced deletion frequencies at the targeted sites (23, 25). These observations suggest that larger deletions tend to increase with the inhibition of NHEJ pathway components. DNA breaks induced in the G1 phase have been reported to be repaired in the S–G2/M phase, which leads to translocations (26).

To address the concerns surrounding the off-target effects of CRIPR/Cas9 editing, in this study, we leverages the unique characteristics of TK6 cells, which can be selected with trifluorothymidine (TFT) because of their ability to sustain deletions larger than 81 bp or substitutions within exon 5 (27). We hypothesized that frequent DNA cleavage by gRNA/Cas9 impairs the ability of cells to repair naturally occurring mutations, potentially leading to erroneous repairs or genomic instability. By synchronizing cell cycles and inhibiting cyclin-dependent kinase 1 (CDK1), we demonstrated that it is possible to mitigate the development of genome-wide mutations and rearrangements induced by genome editing. Furthermore, we explored the role of alternative DNA repair pathways such as those involving zinc finger (ZNF) proteins in suppressing genome instability during CRISPR-Cas editing.

## Results

### Lack of repair through NHEJ pathway increases genome editing efficiency at target site

**Figure 1:**
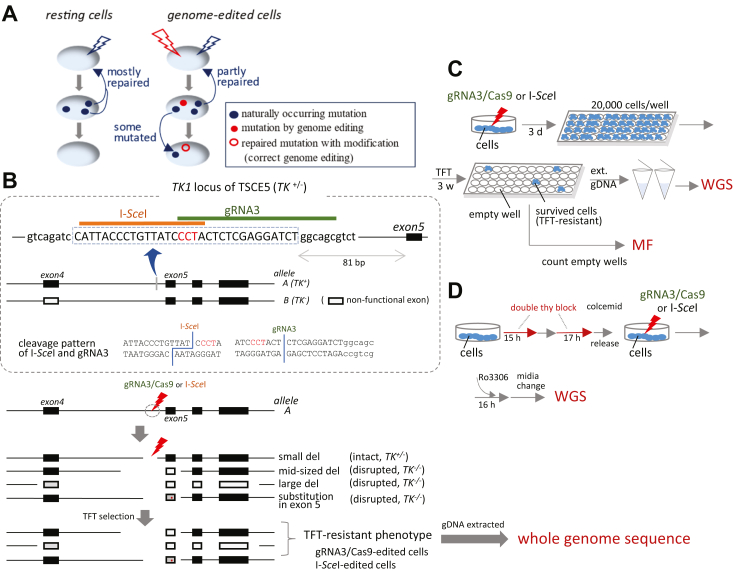
**The surviving cells, with disrupted exons in the allele A of TK1 gene, were analyzed to reveal genome-wide mutations.***A*, relationship between naturally occurring and genome-edited mutations. Cells repair naturally occurring mutations daily. Cells correct artificial DNA cleavage induced by genome editing, such as gRNA/Cas9. Some cells correct both naturally occurring and genome-edited mutations, whereas others repair one or neither. Consequently, genome-edited cells may contain both the desired mutation at the target site of the genome editing and naturally occurring mutations throughout the genome. We wondered whether genome editing increases naturally occurring mutations that need to be repaired. *B*, TSCE5 cells were constructed by inserting a 32-base sequence containing I-*Sce*I recognition site (*dotted square*) at the 81 bp upstream of exon 5 (allele A) of TK6 cells, which have nonfunctional exon 4 in the other allele (allele B). A single guide RNA (gRNA3) was designed between the insertion sequence and the genome of TSCE5 cells. Large deletion involving exon 5 or other exons induced by gRNA3/Cas9 or I-*Sce*I results in functional loss of *TK1* and trifluorothymidine (TFT)-resistant phenotype. Substitutions within exon 5 also disrupts *TK1*. *C*, double-strand breaks (DSBs) was induced at each target site (I-*Sce*I or gRNA3/Cas9). Cells were seeded into 96-well culture plates after a 3-day culture and then selected in the presence of TFT. Cells with TFT-resistant phenotypes can survive, whereas other cells are dead (empty wells). Genomic DNA (gDNA) was extracted from surviving cells (TFT-resistant) with disrupted exons in allele A of *TK1* for whole-genome sequence analysis. Cell culture in 96-well culture plates generated large or small colonies. *D*, similar experiments were performed under synchronous (S–G2/M arrest followed by cyclin-dependent kinase 1 (CDK1) inhibition [S–G2/M] conditions, where double thymidine (d-thy) block and colcemid were used to synchronize cells before genome editing. CDK inhibitor Ro3306 (Ro) was also used to block cell division during genome editing by I-*Sce*I or gRNA3/Cas9 addition (S–G2/M conditions), and cells that survived TFT selection formed one colony. TK, thymidine kinase.

Cells repair naturally occurring mutations every single day. Most mutations are correctly repaired in resting cells. However, we do not know how the induction of artificial DNA breaks affects the repair of mutations that occur naturally throughout the genome. When artificial DNA DSBs are induced by gRNA3/Cas9, cells begin to repair not only naturally occurring mutations but also genome-edited DNA cleavages (Fig. 1*A*). Do cells have the ability to correct all these mutations? If not, the cells will accumulate mutations and we need to find the best way to prevent them from the development of those mutations.

**Figure 2:**
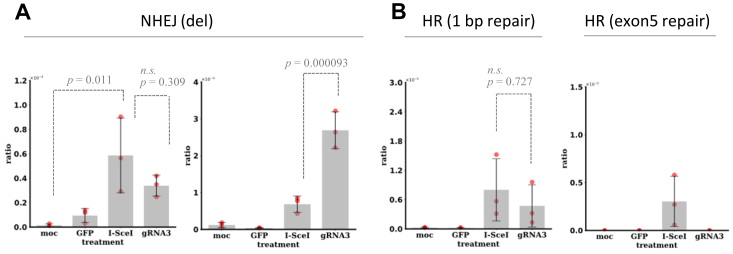
**Thymidine kinase assay was used to selects cells with deletions > 81 bp or mutations in exon 5 using trifluorothymidine in TSCE5 cells, and to select cells repaired by homologous recombination using cytidine and HAT in TSCE2 and TSCE122 cells.** TSCE2 carries 1 bp mutation in exon 5, while TSCE122 lacks 335 bp, including whole exon 5. *A*, mutations in TSCE5 cells detected in nonhomologous end-joining (NHEJ) pathway. Mutant frequencies (MFs) of large deletions (>81 bp) were almost the same in gRNA3/Cas9- and I-*Sce*I–edited [MF = 3.39 *versus* 5.84 (×10^−5^)] under nonsynchronous (non-syn) conditions. In contrast, MF in gRNA3/Cas9-edited cells was much higher than that in I-*Sce*I–edited [27.2 *versus* 8.10 (×10^−5^)] cells under S–G2/M conditions. gRNA/Cas9-edited cells increased the frequency 8.02-fold under S∼G2/M conditions compared to non-syn conditions. Data are from three independent experiments (mean ± SD). *B*, frequencies of HR against 1 bp point mutation (G- > A) at 23rd base from beginning of exon 5 and missing 335 bp were estimated in TSCE2 and TSCE122 cells under non-syn conditions. HR frequencies against 1 bp substitution were similar in I-*Sce*I- and gRNA3/Cas9-edited cells. Repair of missing 335 bp was detected in I-*Sce*I-, but not gRNA3/Cas9-edited cells. All 384 wells (four 96-well plates) were empty after drug selection in TK assay, indicating difficulty of gRNA/Cas9-initiated repair in HR pathway. Data are from three independent experiments (mean ± SD). gRNA, guide RNA.

To address this, we analyzed genome sequences of nonedited cells and edited cells by CRISPR/Cas9. In particular, we focused the genome-edited cells with larger deletions at the target site or with base substitutions in exon 5 because those mutations are derived from lack of the repair through the pathways, such as NHEJ and base/nucleotide excision repair (BER/NER). In this study, we used the cell system consisting of TSCE5 cells (28), with a heterozygous thymidine kinase (TK) genotype (*TK*^*+/−*^, Fig. 1*B*), which survived in the presence of TFT, to acquire cells harboring larger deletions (>81 bp) around the target site or base substitutions within exon 5. Target sites for gRNA3/Cas9 and I-*Sce*I were inserted into the functional allele of TK1 gene (allele A), 81 bp before the exon 5, so that the formation of larger deletions or the occurrence of mutations within exon 5 disrupts the TK1 gene, which leads from *TK*^*+/-*^to *TK*^−/−^ genotype. Methodologically, TFT is converted to TFT monophosphate by the TK1 gene in *TK*^+/−^ cells, which inhibits thymidylate synthase for DNA synthesis. On the other hand, in *TK*^−/−^ cells, TFT is not converted to TFT monophosphate. Therefore, *TK*^−/−^ cells can survive in the presence of TFT. We then scrutinized the genomes of the surviving cells (Fig. 1, *C* and *D*). Whole genomes of the surviving cells were analyzed in detail using next-generation sequencing (NGS). We used three different cell lines. TSCE5 cells were used to analyze mutations derived from the NHEJ pathway. TSCE2 and 122 cells were used for measuring the ratios of DNA repair through the HR pathway. TSCE122 lacks whole exon 5 in TK1 gene, whereas TSCE2 has a point mutation in the exon 5. The repair of whole exon 5 is more difficult than correcting a single nucleotide substitution (Fig. S1). First, we measured the mutant frequency and the repair frequency of gRNA3/Cas9-edited and I-*Sce*I–edited cells using the three different cells. In TSCE5 cells, two cell culture conditions were used: nonsynchronous (hereinafter referred to as non-syn) and synchronous conditions (S∼G2/M arrest followed by CDK1 inhibition, S∼G2/M). Mutant frequencies (MFs) are the occurrence ratios of larger deletions (>81 bp) around the target site or small mutations such as base substitutions within exon 5 of *TK1*. The MFs were 3.39 × 10^−5^ and 5.84 × 10^−5^ in gRNA3/Cas9-edited cells and I-*Sce*I–edited cells, respectively, under non-syn conditions. In contrast, the MFs were 27.2 × 10^-5^ and 8.10 × 10^−5^ in gRNA3/Cas9-edited cells and I-*Sce*I–edited cells, respectively, under S ∼ G2/M conditions (Fig. 2*A*). The frequency of gRNA3/Cas9-edited cells increased 8.02-fold under S∼G2/M conditions compared to non-syn conditions. This result is consistent with reports published so far (29, 30, 31, 32, 33). The results of the measurement of repair frequencies, which are the ratios of DNA repair through the HR pathway, in TSCE2 cells showed values of 0.47 × 10^−5^ and 0.80 × 10^−5^ in gRNA3/Cas9-and I-*Sce*I–edited cells, respectively. In TSCE122 cells, the repair of whole exon 5 was detected only in I-*Sce*I–edited cells with a repair frequency of 0.27 × 10^−5^. This is probably due to the difference in the DSB pattern of DNA, that is, blunt ends for gRNA3/Cas9 and staggered ends for I-*Sce*I. Alternatively, the longer binding at the target site of gRNA3/Cas9 after DNA cleavage can be another cause.

### Genome editing increased genome-wide mutations, which are suppressed under S–G2/M conditions

**Figure 3:**
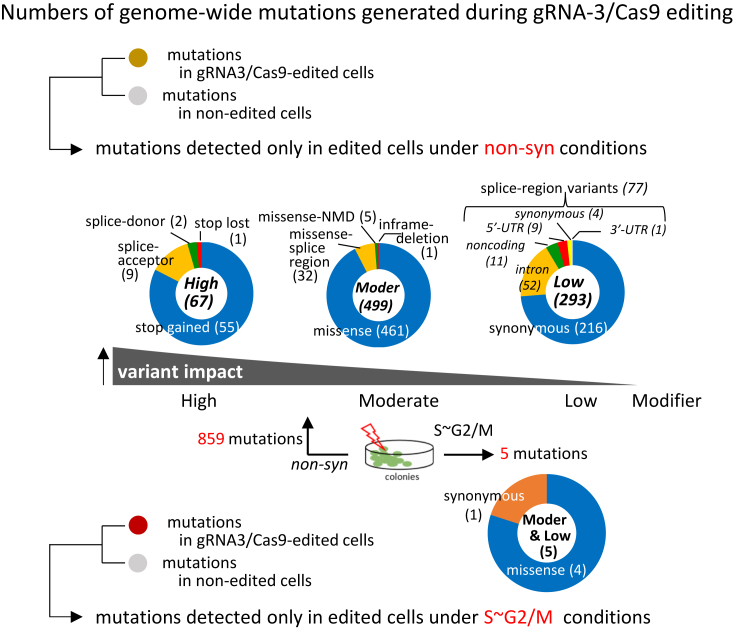
**Comparison of genome-wide mutations such as indels and base substitutions induced by gRNA3/Cas9 editing in TSCE5 cells under nonsynchronous and S–G2/M arrest, followed by cyclin-dependent kinase 1 inhibition (S–G2/M) conditions.** Genome-wide mutations were extracted by comparing nonedited control and gRNA3/Cas9-edited cells, using Mutect2 software in GATK. The vcf files were functionally annotated using Ensembl variant effect predictor (VEP, release 103, GRCh38.p13). Annotated files were filtered to remove variants marked with the impact “modifier” and to delete redundant variants. Resulting files were manually inspected, and variants were defined as “high,” “moderate,” and “low.” High-impact variants are “stop codon gained/stopped” and “splicing donor/acceptor.” Descriptions of variant consequences follow calculated variant consequences in Ensembl VEP. Low-impact variants were synonymous or splice-region variants, the latter containing intron, noncoding, UTR, and synonymous variants (*italicized*). gRNA3-/Cas9–edited cells generated 859 mutations under non-syn conditions, whereas cells exhibited only five mutations under S–G2/M conditions. Result shows variants in gRNA3/Cas9-edited cells were significantly reduced under S–G2/M conditions. Similar results were obtained in I-*Sce*I–edited cells (Fig. S5). Data are from two independent experiments. gRNA, guide RNA.

When DNA DSBs are induced by gRNA3/Cas9, cells need to correct the additional DNA lesions. To investigate the effect of DNA DSBs by gRNA3/Cas9 on the repair of naturally occurring mutations, we analyzed the genomes of nonedited TSCE5 cells, gRNA3/Cas9-edited TSCE5 cells and I-*Sce*I–edited TSCE5 cells and compared the whole sequences of genome-edited cells with the sequence of nonedited cells to extract newly emerged mutations. Mutations such as base substitutions and indels were also investigated genome-wide and extracted using the GATK/Mutect2 software (Broad Institute, https://gatk.broadinstitute.org/hc/en-us). The mutations that occurred only in gRNA3/Cas9 or I-*Sce*I–edited cells are summarized in Figure 3, Tables S1, and S2, showing cellular functional impacts (high-, moderate-, and low-impact variants) using the Ensembl-variant effect predictor (VEP, ver.103).

A total of 51,756 mutations (*incl*. modifier impact mutations that do not affect cellular functions) with redundancy were detected in gRNA3/Cas9-edited cells (*versus* nonedited TSCE5 cells). Among them, 859 nonredundant mutations that can affect cellular functions were observed under non-syn conditions in gRNA3/Cas9-edited cells. In contrast, under S∼G2/M conditions, only five nonredundant mutations out of 807 mutations were observed to affect cellular function (Fig. 3). During these experiments, the synchronous state of the cell cycle was checked immediately before genome editing (Fig. S2). In I-*Sce*I–edited cells, 97,120 mutations were detected, including 1665 that can affect cellular functions under non-syn conditions. In contrast, only nine out of 1233 mutations were observed to affect cellular function under S∼G2/M conditions (Fig. S3*A*).

Simultaneously, our examination of naturally occurring mutations in the resting test cells showed that TSCE5 cells harbored 16 mutations that could affect their cellular functions (Fig. S4), whereas retinal pigment epithelial (RPE)-1 cells, carrying an intact *TP53* gene similar to TSCE5, had 29 to 35 mutations. Therefore, the 859 mutations in gRNA3/Cas9-edited TSCE5 cells under non-syn conditions were much higher than the 16 mutations in resting TSCE5 cells. Among the genetic mutations detected in the gRNA3/Cas9-edited and I-*Sce*I–edited cells under non-syn conditions, 113 genes, including *ATM* and *TP53BP1*, were commonly observed (Fig. S3*B*). Under S–G2/M conditions, genome-wide mutations caused by genome-editing tools were effectively suppressed.

### Mutation signatures in gRNA3/Cas9-edited cells under two culture conditions

Cells repair naturally occurring mutations caused by oxidation, alkylation, and replication error (34, 35), such as the formation of 8-oxoguanine, *O*^6^-methylguanine and pyrimidine dimer, and oxidative deamination of cytosine. These mutations are corrected by BER, NER, and mismatch repair (MMR).

**Figure 4:**
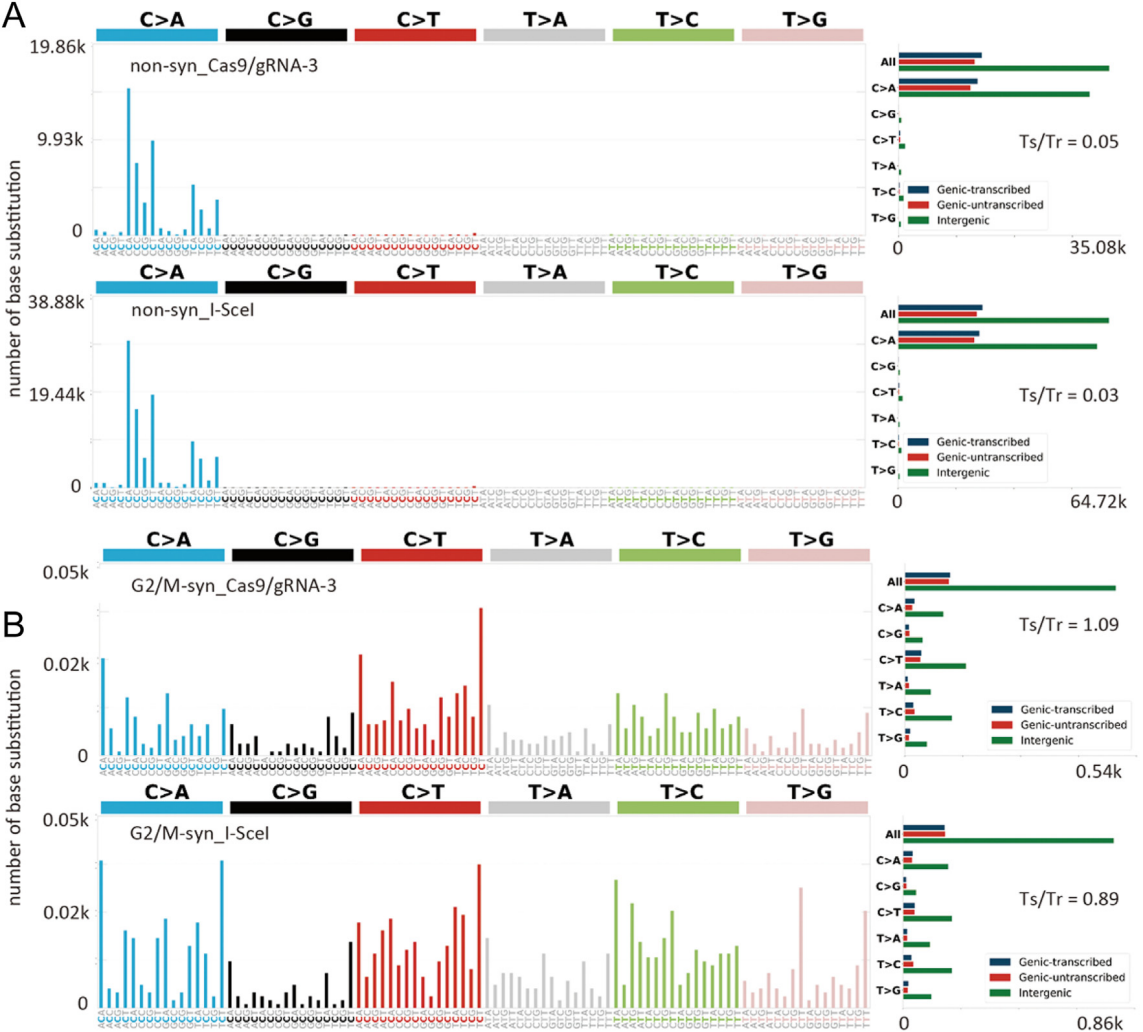
**Single-base substitution signatures generated in genome-edited cells under nonsynchronous and S–G2/M arrest followed by CDK1 inhibition (S–G2/M) conditions.***A*, SBS profiles of gRNA3/Cas9- and I-*Sce*I–edited cells under non-syn conditions. Most substitutions were C:G to A:T conversions (transversion, Tv). Ratios of transition to transversion (Ts/Tv) were 0.03 to 0.05. *B*, SBS profiles under S–G2/M conditions. Substitutions were much lower than under asynchronous conditions. No distinct substitution patterns were observed. Ts/Tv were 1.09 and 0.89. gRNA, guide RNA.

Mutations were detected in gRNA3/Cas9-edited and I-*Sce*I–edited cells under non-syn conditions. To determine whether genome editing caused specific mutation patterns, we examined the mutation signatures of these cells. Single and doublet base substitution signatures were analyzed using SigProfiler Matrix Generator tools. Numerous single-base substitutions were generated in gRNA3/Cas9-edited and I-*Sce*I–edited cells under non-syn conditions (Fig. 4*A*). Most base substitutions were C:G to A:T transversion (Fig. 4*A*, *right panel*). Ratios of transition to transversion (Ts/Tv) were 0.05 and 0.03 in gRNA3/Cas9-edited cells I-*Sce*I–edited cells, respectively. Replication errors and DNA oxidation/alkylation could cause the accumulation of C:G to A:T transversion, such as 8-oxoguanine. Similar signatures to our result were reported in patients with mutations of *POLD1* and *MUTYH*, defective DNA MMR/BER, and with the habit of tobacco smoking producing reactive oxygen species in COSMIC catalog of somatic mutations in cancer (ver. 96). Mutations in *POLD1*, which is involved in DNA replication and repair, and *MUTYH*, which is involved in BER, were not observed in gRNA3/Cas9-and I-*Sce*I–edited cells (Table S1). Numerous C:G to A:T transversions were not caused by mutations in these genes. In contrast, under S–G2/M conditions, Ts/Tv was 1.09 and 0.89 in gRNA3/Cas9- and I-*Sce*I–edited cells, respectively (Fig. 4*B*) and these ratios are common.

### Genome rearrangements are suppressed in cells under S∼G2/M conditions

**Figure 5:**
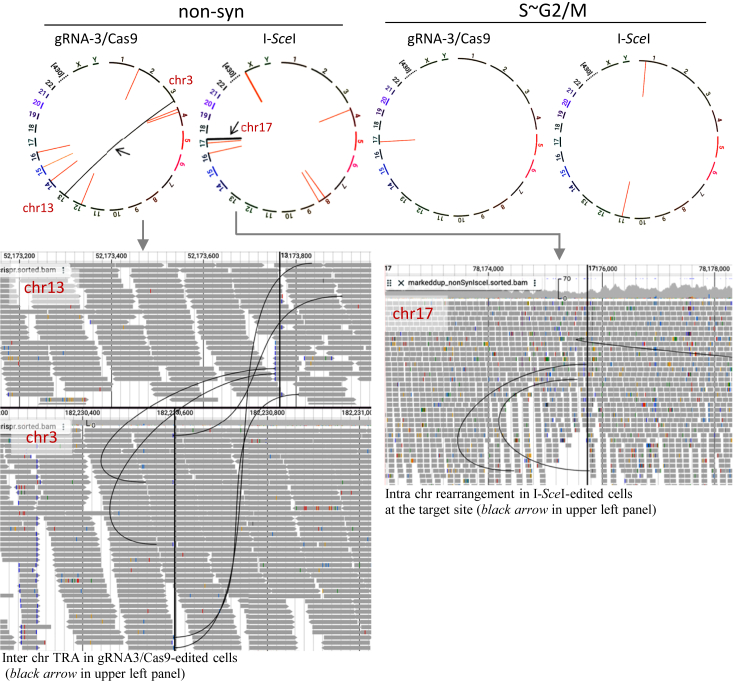
**Structural variations detected in genome-edited TSCE5 cells.** Circus plots showing one interchromosomal translocation between chromosome 3 (chr3) and chr13 and seven intrachromosomal rearrangements in gRNA3/Cas9-edited cells. Seven intrachromosomal rearrangements were observed in I-*Sce*I–edited cells under non-syn conditions (*left panel*). In S–G2/M conditions, one and two genome rearrangements were detected in gRNA3/Cas9- and I-*Sce*I–edited cells, respectively (*right panel*). Structural variants (SVs) were visualized using JBrowse2. gRNA, guide RNA.

Structural variants (SVs) of gRNA3/Cas9-edited and I-*Sce*I–edited cells were analyzed under non-syn and S∼G2/M conditions. Under non-syn conditions, one interchromosomal translocation between chromosome (chr) 3 and chr 13 and seven intrachromosomal rearrangements were detected in gRNA3/Cas9-edited cells (Fig. 5). In contrast, under S∼G2/M conditions, only one intrachromosomal rearrangement was observed at chr 17. In addition, in I-*Sce*I–edited cells, seven and two intrachromosomal rearrangements were detected under non-syn and S∼G2/M conditions, respectively.

The number of genome rearrangements was lower under S–G2/M conditions than it was under non-syn conditions in both gRNA3/Cas9-edited and I-*Sce*I–edited cells. (Table S3). Genome rearrangements were suppressed under S–G2/M conditions. Moreover, the investigation of copy number variations (CNVs) showed no alterations or oscillations in the gRNA3/Cas9-edited cells under non-syn and S–G2/M conditions (Fig. S6).

### FISH analysis reveals no chromosome aberrations in gRNA3/Cas9-edited cells

**Figure 6:**
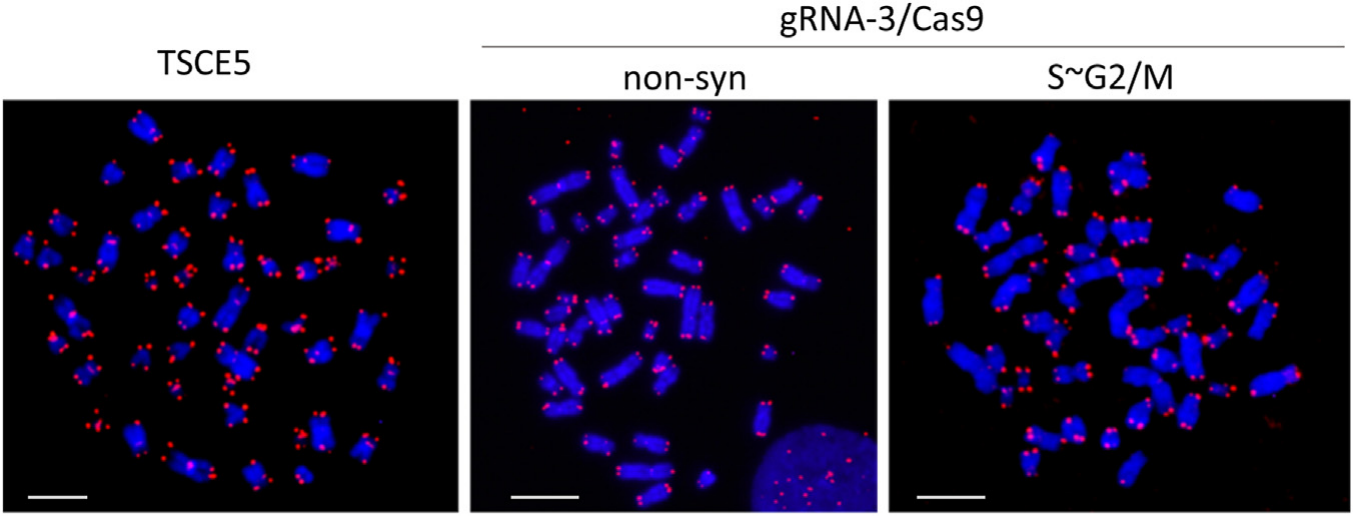
**Chromosome aberrations in gRNA3/Cas9-edited cells.** FISH analysis of gRNA3/Cas9-edited cells under non-syn and S–G2/M conditions. Chromosomal cleavage, fusion, and micronucleus formation were not observed in gRNA3/Cas9-edited TSCE5 cells. Experiment was repeated twice. Representative images are shown. The scale bar represents 10 μm. gRNA, guide RNA.

The FISH analysis to further confirm the absence of structural chromosomal rearrangements in gRNA3/Cas9-edited cells revealed no chromosomal breaks, fusions, and micronuclei under non-syn and S–G2/M conditions (Fig. 6). This result is consistent with the findings of the genome analysis (Fig. 5). This indicates that genome editing under S∼G2/M conditions is less harmful for cells.

### Cell cycle synchronization with CDK1 inhibition activates alternative DNA repair genes

**Figure 7:**
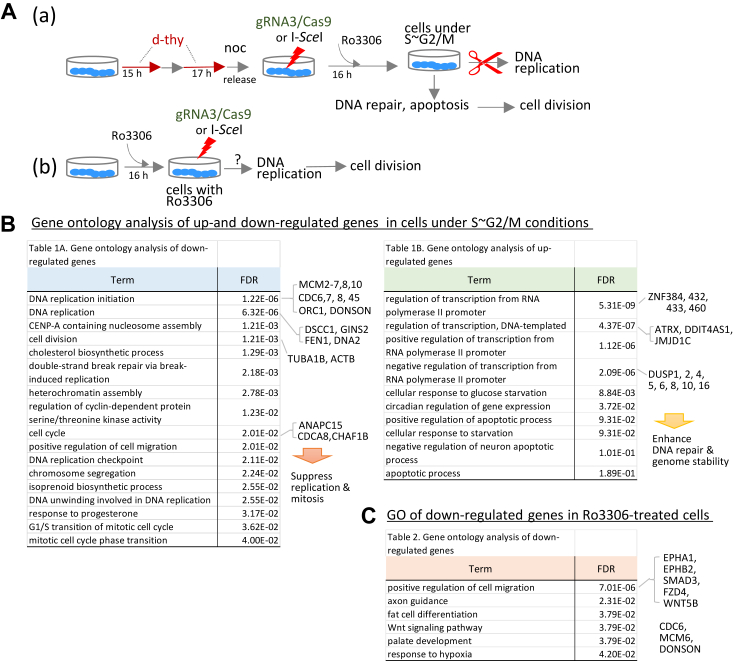
**Gene profiles of cells under S–G2/M conditions or cells treated with Ro3306 alone.** Transcriptome analysis was performed using two different synchronization methods: double thymidine block (d-thy)/nocodazole (noc) treatment, followed by Ro3306 treatment and Ro3306 alone. Total RNA was extracted from control cells, d-thy/noc/Ro3306-treated cells (*i.e.*, S–G2/M conditions), and Ro3306-treated cells. Numerous genes were upregulated or downregulated in d-thy/noc/Ro3306-treated cells. Genes were extracted under conditions of log_2_FoldChange >1 and false discovery rate (FDR) < 0.05. *A*, d-thy/noc followed by Ro3306 treatment comprehensively suppressed genes related to DNA replication and cell division and activated genes for DNA repair (a). Ro3306 treatment suppressed cell division but did not block DNA replication initiation in DNA-damaged cells (b). Cell cycle synchronization suppressed genome-wide mutations. Transcriptome data were obtained two in *A* (a) and four in *A* (b) independent experiments. *B*, gene ontology (GO) analysis of cells under S–G2/M conditions revealed downregulation of genes associated with DNA replication and cell cycle. Expression of minichromosome maintenance 2 to 7 (*Mcm2–7*), cell division cycle 6 to 7 (*CDC6–7*), *CDC45*, GINS complex subunit 2 (*GINS2*), origin recognition complex 1 (*ORC1*), and downstream neighbor of Son (*DONSON*) genes required for Cdc45–Mcm2 to 7–GINS (CMG) complex formation and DNA replication initiation was significantly suppressed in cells under S–G2/M conditions. In contrast, zinc finger protein 384 (ZNF384), ZNF432, and dual specificity phosphatase (DUSP) were activated in these cells. ZNF384 and ZNF432 are considered to be involved in repair of ssDNA cleavage. DUSP6 modulates DNA damage response. *C*, GO analysis of Ro3306-treated cells. Few genes associated with DNA replication, including *CDC6*, *MCM6*, and *DONSON*, were downregulated. Gene expression profiles of Ro3306-treated cells were distinct from those of cells under S–G2/M conditions No significantly upregulated terms were found in GO analysis.

We conducted a transcriptome analysis to identify the genes involved in the suppression of numerous genome-wide mutations. Furthermore, to elucidate the role of cell cycle synchronization and CDK1 inhibition in this process, the analysis included an additional culture condition involving Ro3306 treatment alone (Fig. 7*A*). Gene ontology (GO) analysis of the transcriptome data using the database for annotation, visualization, and integrated discovery revealed a reduction in the expression of genes associated with DNA replication and initiation. Gene expression profiles in gRNA3/Cas9-edited cells under S–G2/M conditions compared to nonedited control cells (Fig. S4).

These genes, including minichromosome maintenance 2 to 7 (*MCM2-7*), *MCM8-9*, cell division cycle 6 (*CDC6*), *CDC45*, origin recognition complex 1, and GINS complex subunit 2, and the protein downstream neighbor of Son (*DONSON*), were significantly reduced in gRNA3/Cas9-edited cells under S–G2/M conditions (Fig. 7*B*, left table). The functions of these molecules are well documented in the literature (36, 37, 38, 39, 40, 41, 42). Conversely, the expression of genes involved in DNA repair and damage responses, such as ZNF384, ZNF432, and dual specificity phosphatase (DUSP) genes, was significantly upregulated in gRNA3/Cas9-edited cells under S–G2/M conditions (43, 44, 45, 46, 47, 48). Additionally, O-6-methylguanine methyltransferase (MGMT) was activated (log2FC = 2.58, false discovery rate = 1.5 × 10^−4^), and the gene expression of xeroderma pigmentosum complementation group C (XPC), which interacts with unpaired DNA bases, was significantly upregulated (log2FC = 1.83, false discovery rate = 3.4 × 10^−4^). These three genes are involved in BER and NER. The result suggests that ZNF and DUSP proteins enhance DNA repair capacity in gRNA3/Cas9-edited cells under S∼G2/M conditions where DNA replication is suppressed.

Cells treated with Ro3306 alone showed no changes in these genes. No significant GO terms were observed in cells treated with Ro3306 alone. Ro3306, which inhibits CDK1/cyclin B1 activity by binding to the ATP pocket of CDK1, consequently prevents mitosis (49).

## Discussion

**Figure 8:**
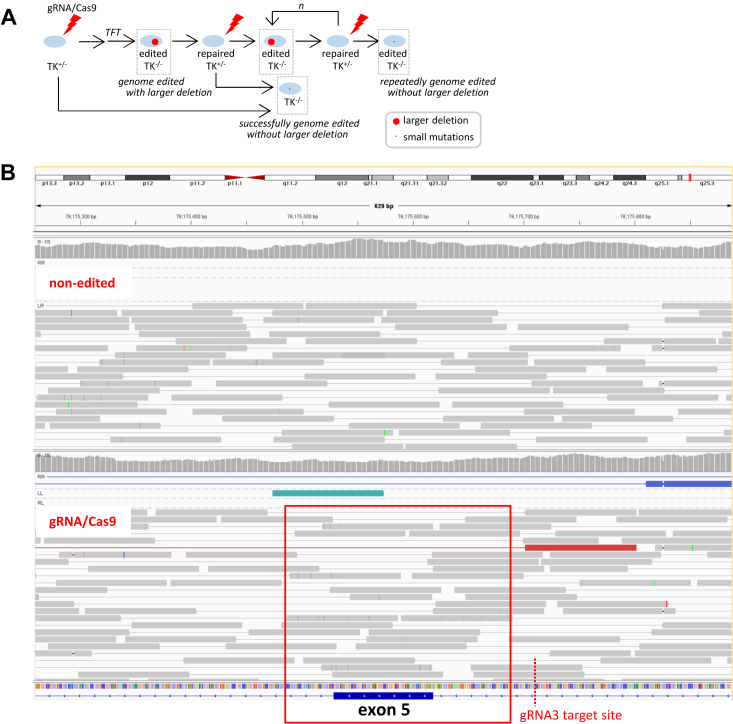
**TSCE5 cells were edited by gRNA3/Cas9 and selected with TFT.***A*, phenotype of resulting gRNA/Cas9-edited cells was derived from homozygous thymidine kinase genotype (*TK*^*−/−*^), containing cells with disrupted exon 5. Some cells had deletions > 81 bp at target site, whereas others had small mutation in exon 5 of thymidine kinase gene (*TK*). *B*, gRNA/Cas9-edited cells induced mutations within exon 5 of *TK*, visualized using Integrative Genomics Viewer (IGV). gRNA, guide RNA; TFT, trifluorothymidine.

TSCE5 cells, used to investigate whether artificial DNA cleavage induces genome-wide mutations, exhibited deletions greater than 81 bp, as well as small mutations such as base substitutions within exon 5. Genome editing tools initiate DNA breaks at the target site, and while most of the DNA breaks are repaired, the breaks are then induced again. Repeated breaks and repairs can attenuate DNA repair ability, preventing the correction of naturally occurring mutations. (Fig. 8).

This study provides two key insights into the behavior of genome-wide mutations during gRNA3/Cas9 editing. First, although gRNA3/Cas9 editing induces genome-wide mutations and increases the frequency of C:G to A:T transversions under standard cell culture conditions (non-syn conditions), these mutations were suppressed under S-G2/M conditions. Second, the combination of S–G2/M arrest and CDK1 inhibition using Ro3306 (S–G2/M conditions), rather than Ro3306 treatment alone, enhances the expression of emergent DNA repair genes, such *as ZNF384*, *ZNF432*, and *DUSP6*. To address the challenges associated with the development and accumulation of genome-wide mutations during genome editing, we analyzed the genome sequences, mutation signatures, and SVs of non-edited and genome-edited cells using gRNA3/Cas9 and I-*Sce*I.

Cells are well known to repair over 50,000 naturally occurring mutations daily to prevent accumulation of DNA damage and ensure survival (35, 50). These naturally occurring mutations are caused by oxidation, alkylation, and replication errors, such as the formation of 8-oxoguanine, *O*^6^-methylguanine, and pyrimidine dimers and oxidative deamination of cytosine. These mutations are corrected by BER, NER, and MMR. When DNA double-stranded breaks are induced by gRNA3/Cas9, the cells correct for additional DNA lesions.

In the resting state, TSCE5 cells retain robust DNA repair capabilities, maintaining a low mutation presence (16 mutations) (Fig. S4). However, following artificial DNA double-strand cleavage by gRNA3/Cas9, these cells initiate repair processes for both the induced and naturally occurring mutations. Repeated DNA cut and repair at the gRNA3/Cas9 target site lead to ongoing cycles of cleavage and repair in primary or p53-competent cells (3, 51). Under non-syn conditions, this cycle contributes to the accumulation of genome-wide mutations, with 859 mutations observed in gRNA3/Cas9-edited cells, compared to only 16 in nonedited TSCE5 cells. However, under S-G2/M conditions, the mutation count drops dramatically to just five (Figs. 3 and S3).

Mutation signature analysis highlights a high incidence of C to A transversions under non-syn conditions, which is likely due to the build-up of oxidized and alkylated DNA bases, such as 8-oxoguanine and O-methylguanine (Fig. 4*A*), which are typically repaired through BER processes. Conversely, such transversions are rare under S-G2/M conditions.

The comprehensive transcriptome analysis conducted on RPE-1 cells, which have an intact TP53 gene. This is targeted at elucidating the genetic mechanisms underlying the suppression of genome-wide mutations, including determining the role of cell cycle synchronization and CDK1 inhibition in this context. To achieve this aim, we included an additional culture condition involving Ro3306 treatment alone.

This analysis reveals a significant suppression of genes critical for DNA replication initiation, such as *CDC45*, *MCM2-7*, and GINS complex subunit 2, which are essential for Cdc45–Mcm2-7–GINS complex formation, along with *DONSON*, origin recognition complex *1*, and *CDC6*, which were significantly downregulated in gRNA3/Cas9-edited cells under S-G2/M conditions (Fig. 7*B*, left). The functions of these molecules are well-documented in the literature (36, 37, 38, 39, 40, 41, 42). No such downregulation was observed in cells exposed to Ro3306 treatment alone, except for *CDC6, MCM6*, and *DONSON*, which suggests that Ro3306 suppressed DNA replication and initiation, although the inhibition of mitosis, may not be critical (49).

In contrast, ZNF proteins are significantly upregulated under S-G2/M conditions (Fig. 7*B*, right). ZNF384, a member of the C2H2 family of ZNF proteins, plays a crucial role in DNA repair by binding to DNA break ends and promoting Ku70/80 assembly. The unfolding of chromatin, facilitated by PARP1, enhances the DNA-binding capacity of ZNF384 at DNA damage sites (48). ZNF432 binds to ssDNA and impedes DNA resection. These ZNF proteins are integral to both NHEJ and HR repair pathways, underscoring their significance in genome stability maintenance (47).

Furthermore, the activation of genes associated with NER and BER, such as significantly upregulated XPC and MGMT genes, suggests an enhanced capacity for repairing naturally occurring mutations under S-G2/M conditions. Additionally, proteins such as DUSP6 play a pivotal role in modulating the DNA damage response, contributing to the heightened repair capabilities observed (43, 44). These results suggest that ZNF, DUSP, MGMT, and XPC enhance the DNA repair capacity in gRNA3/Cas9-edited cells under S–G2/M conditions.

In conclusion, this study demonstrates that gRNA3/Cas9 editing not only induces genome-wide mutations but also reveals the conditional dynamics of their suppression. Under non-syn conditions, the significant increase in genome-wide mutations, particularly C:G to A:T transversions, highlights the vulnerability of genome stability to the editing processes. However, under S-G2/M conditions, where cell cycle synchronization in the S-G2/M phase and CDK1 inhibition are combined, these mutations dramatically reduce, and this is accompanied by the upregulation of DNA repair genes such as ZNF384, ZNF432, and DUSP6. These findings underscore the critical role of cell cycle control and specific DNA repair mechanisms in mitigating the unintended consequences of genome editing. Furthermore, cell cycle control with CDK1 inhibition enhances the precision and safety of CRISPR technologies in genetic research and therapeutic applications.

## Experimental procedures

### Cells

TSCE2, TSCE5, and TSCE122 cells were kindly provided by Masamitsu Honma and Manabu Yasui of the Division of Genetics and Mutagenesis at National Institute of Health Sciences. This study did not generate any new reagents.

### Cell culture and cell cycle synchronization

TSCE5 cells, which were generated from TK6 cells by integrating the I-*Sce*I site into intron 4 (81 bp upstream of exon 5) of *TK1 via* HR, were used in this study. Additionally, we designed gRNA for CRISPR/Cas9 (designated as gRNA3/Cas9) in the region between the I-*Sce*I insertion sequence and the genomic sequence of TSCE5 cells. Briefly, the cells were maintained in RPMI 1640 medium and those with a dysfunctional *TK1* gene were selected using the TK assay described in the next section. The target sites for gRNA3/Cas9 and I-*Sce*I were inserted into the functional allele of the *TK1* gene (allele A), 81 bp before exon 5, to ensure that the formation of larger deletions > 81 bp around the target sites or the occurrence of mutations within exon 5 disrupted the *TK1* gene rendering it dysfunctional and knocked out as described in Figure 1, leading to the *TK*^*−/−*^ genotype. Subsequently, the cells transformed into the *TK*^*−/−*^ genotype survived in the presence of TFT.

To synchronize cells in the S∼G2/M phase, they were cultured with 1.5 mM of thymidine for 15 to 18 h twice with a 10-h release (double thymidine block [d-thy]) and maintained for 2 h with colcemid (0.1 μg/ml, Gibco, Thermo Fisher Scientific). Plasmids encoding I-*Sce*I (pCBASceI, #26477) or gRNA3/Cas9 (p x 459, #48139; both from Addgene) were electroporated into the cells for genome editing. Ro3306 (5 μM, Sigma-Aldrich) was added to the S∼G2/M-synchronized cells after the electroporation, followed by incubation overnight. Cell cycle status was visualized using the cell-clock cell cycle assay kit (Biocolor). D-thy alone does not synchronize the cell cycle and, therefore, this was followed by a short colcemid treatment (because prolonged exposure is cytotoxic) to synchronize the cell cycle at the S–G2/M phase. This combination treatment is a better method for synchronizing TSCE5 cells.

TSCE2 and TSCE122 cells repaired *via* the HR pathway were selected in the presence of 2′-deoxycytidine and hypoxanthine-aminopterin-thymidine (cytidine and HAT). Human telomerase reverse transcriptase RPE cells were used for transcriptome analyses. RPE-1 cells were maintained in Dulbecco's modified Eagle's medium/F-12 in the presence of hygromycin B (10 μg/ml, InvivoGen). To synchronize cells in the S∼G2/M phase, they were cultured twice with 1.5 mM of thymidine for 14 to 19 h each time with a 9-h release (d-thy) and maintained for 5 h with nocodazole (noc, 50 ng/ml, Sigma-Aldrich). After genome editing, Ro3306 was added to suppress cell division and the cells were maintained for 16 h.

### TK assay

The TK assay was performed using an *in vitro* mammalian cell gene mutation test with the *TK* gene (OECD validated test No. 490). The genome editing efficiency of TSCE5 is shown in Fig. S1*B*. TSCE5 cells were treated with cytidine and HAT for 3 days to completely suppress background *TK*^−/−^ cells, electroporated with plasmid encoding gRNA3/Cas9 or I-*Sce*I (3 μg) using the Amaxa Nucleofector 2b transfection device (program No. A-023, Lonza), and then cultured for 72 h in T-75 flasks. The cells were then seeded in 96-well plates at a density of 20,000 cells/well in the presence of TFT (2 μg/ml) and cultured for another 10 days. The number of wells without colonies (Ew) was counted to calculate the MF. In parallel, another set of electroporated cells was seeded into two 96-well plates at a density of one cell/well, and the number of wells without colonies (Ew') was calculated.

The MF was calculated as -ln (Ew/Tw)/20,000 ÷ -ln (Ewʹ/Twʹ)/1, where Ew and Tw indicate the number of wells without any colonies and the total number of wells, respectively. After TFT selection, the cells formed either small or large colonies, which indicated a potential mutation at a locus involved in cell growth or normal cell growth, respectively. Surviving *TK*^−/−^ cells were selected and cultured for several days to extract genomic DNA for NGS analysis. The same experiment was performed in S–G2/M-synchronized TSCE5 cells in the presence of Ro3306, which was added after genome editing and the cells were incubated overnight. The medium was then discarded, and fresh medium was added.

### Amplicon sequencing

Low-frequency mutation patterns were examined around the target site of SpCas9/gRNA3 using the amplicon sequence technique. A 10 kb region was amplified using PrimeSTAR GXL DNA polymerase (Takara Bio), and the products were purified using the NucleoSpin gel and PCR clean-up kit (Takara Bio). The purified amplicons (5–10 kb) were sequenced as 151 bp pair-end reads using the iSeq 100 system (Illumina) to obtain 12,000 coverages.

After checking the quality of the reads and trimming the reads using the FastQC (ver. 0.11.9) and Trim Galore (ver. 0.6.7) programs, respectively, the reads were mapped using the BWA-MEM (ver. 0.7.17, r1188) program. PCR duplicates were removed using the Picard software (ver. 2.26, https://broadinstitute.github.io/picard/). As a reference sequence, we used 10 kb of the GRCh38/hg38 sequence centered on the target sequence with insertion of the I-*Sce*I site. CRISPResso2 (ver.2.0.31) was used to identify mutations at the target site of SpCas9/gRNA3 (Fig. S1*B*). SVs were analyzed using Manta (Illumina, ver.1.6.0, https://github.com/Illumina/manta).

### Genome sequence analysis

Whole genomes of the surviving cells were analyzed in detail using NGS. The mutation ratio, size, and patterns, as well as complex genome rearrangements, were investigated in nonedited control cell and gRNA3/Cas9-and I-*Sce*I–edited cells under the two cell culture conditions, non-syn and S–G2/M.

For the analysis, genomic DNA was extracted using the Qiagen blood and tissue kit (Qiagen), and all samples were sequenced using the Illumina Hiaseq 2500 sequencing system. The read coverage was approximately 40× and all bioinformatic analyses were conducted using a Linux operating system (Ubuntu, ver. 20.04) and the R software package (ver. 4.1.2 and later, https://www.r-project.org/). Genome sequences were analyzed and variants present only in gRNA3/Cas9- and I-*Sce*I–edited cells were extracted by comparing the control (plasmid alone) with the genome-edited samples.

Genome sequences were analyzed using the GATK program (ver. 4.1.9.0 and 4.2.2.0) with the best practice workflow. During the analysis, reads sequenced in different lanes of the flow cells were separately handled to build unmapped and aligned binary alignment map (BAM) files, and the resulting two BAM files were merged at the MarkDuplicates step in the Picard program (ver. 2.25). Paired-end reads (150 bp × 2) were aligned to the human reference genome (GRCh38/hg38 for GATK, Google Cloud Bucket) using the BWA-MEM program (ver. 0.7.17, r1188) with the M option. PCR duplicates were marked using MarkDuplicates after the aligned and unaligned BAM files were merged. MergeBamAlignment was run using the options “MAX_INSERTIONS_OR_DELETIONS −1,” “-PRIMARY_ALIGNMENT_STRATEGY MostDistant,” and “CLIP_ADAPTOR false.” The prepared clean BAM files were analyzed using GATK/Mutect2, followed by FilterMutectCalls in GATK to detect somatic variants, such as small substitutions, deletions, and insertions. Mutect2 was run with the option “af-of-alleles-not-in-resource −1.0,” where the tool dynamically adjusts the parameters for an appropriate mode. The af-of-alleles-not-in-resource option was tested at 0.000025 (previously recommended) for comparison; however, both results were the same. The impacts of variants detected were evaluated by the Ensembl-VEP (release 103).

Mutational signatures, including all types of somatic mutations, were analyzed using the SigProfilerMatrixGenerator (COSMIC, Wellcome Sanger Institute, ver.3.2).

SVs and CNVs were analyzed using the following pipelines: SvABA (Broad Institute, ver.1.1) (52) for SVs and ModelSegments CNV Workflow in GATK for CNVs. The software for SV detection was run in somatic modes; for example, the option of -a somatic_run_mode2 in SvABA. The filter-passed SVs were subjected to further analyses. The extraction of passed variants was conducted using SnpEff and SnpSift (ver.5.1). For CNVs, read counts were collected and denoised to generate ModeldSegments file. The CNV results were plotted using the PlotDenoisedCopyRatios tool. Delly (EMBL, ver.0.8.9) and Manta (Illumina, ver. 1.6) for SV analysis were used for comparison.

Circos plots were constructed using the R package Biocircos (ver.0.3.4) (53). R scripts and packages were executed using R studio (Build 443). R was also used for general data handling, such as removing redundant data from Ensembl-VEP and extracting specific characters from the DataFrame, including the extraction of G from atG and Gca.

Mutational signatures, including all types of somatic mutations, were analyzed using the SigProfilerMatrixGenerator (COSMIC, Wellcome Sanger Institute, ver.3.2).

Long-read sequences were analyzed using the PromethION (Oxford Nanopore). The Guppy program(ver.4.4.1) was used to convert fast5 filles to fastq files, which were trimmed with the options of -q 9 and headcrop 75 using Nanofilt (ver.2.8.0). The trimmed reads were aligned to the reference using Minimap2 (ver.2.24) (54). Sniffles (ver.2.0.7) (55) with the options of --minsupport 5, --minsvlen 50, –nongermline were used for SV calls. Somatic variants were obtained by subtracting variants in the control sample from genome-edited samples using bcftools (ver.1.12). The NGS reads were visualized using the Integrative Genomics Viewer (ver.2.13.2) (56) and JBrowse2 (57) were used.

### Transcriptome analysis

Transcriptome analysis was performed for RPE-1 cells under two different synchronization conditions: d-thy/noc treatment followed by Ro3306 treatment or Ro3306 alone. The data were compared with those from non-syn control cells. Total RNA was extracted from control cells and d-thy/noc/Ro-treated cells (*i.e.*, S∼G2/M conditions) or Ro-treated cells using the RNeasy mini kit (QIAGEN). After a quality check of RNAs (RIN>7) using the Bioanalyzer (Agilent), complementary DNA samples were prepared for RNA-seq using the SMART-Seq v4 Ultra Low Input RNA kit. RNA-seq was performed using the NovaSeq 6000 sequencing system (Illumina). The sequence reads were mapped to GRCh38 using the DRAGEN Bio-IT platform (ver.3.6.3). Expression levels are shown as transcripts per million, and genes with more than a 2-fold change between the control and genome-edited groups were extracted. The experimental conditions were the same as those described above. Functional annotation was performed using the database for annotation, visualization, and integrated discovery tool (LHRI) (58). PANTHER was also used for GO analysis to compare the two results (59).

### FISH analysis

FISH was performed according to the Agilent FISH protocol. Briefly, cells were harvested, gently mixed with 0.075 M potassium chloride, fixed with cold methanol–acetic acid (3:1) solution, and stored overnight at −20 °C. The following day, the cells were resuspended, dropped onto glass slides soaked in cooled ethanol, and dried overnight. The slides were then fixed with 4% formaldehyde; washed twice; sequentially dehydrated in 70%, 85%, and 100% ethanol for 1 min each; and then dried.

The slides were subsequently coverslipped by placing then on a cover glass with a hybridization mix containing the TelC-Cy3 telomere probe (Cat# F1002, PANAGENE) and then turning the slides over. The glass slides were placed on a hot plate, heated at 80 °C for 5 min, and then placed on a paper towel for 1 h. The slides were then incubated in FISH wash buffer (Agilent), heated at 73 °C for several minutes, dried with ethanol, and mounted using Pro Long Gold (Invitrogen, Thermo Fisher Scientific) with 4′,6-diamidino-2-phenylindole.

## Data availability

Our dataset used for analysis is available at the DNA Data Bank of Japan (DDBJ, PRJDB18589). We can also provide bam formatted files upon request. Reference human genome sequence was obtained from GATK resource bundle at https://console.cloud.google.com/storage/browser/genomics-public-data/resources/broad/hg38/v0/Homo_sapience_assembly38.fasta.

## Conflicts of interest

The authors declare that they have no conflicts of interest with the contents of this article.
