# Supplementary material for: Cell cycle arrest combined with CDK1 inhibition suppresses genome-wide mutations by activating alternative DNA repair genes during genome editing
Source: J Biol Chem. 2024 Aug 17;300(9):107695. doi: 10.1016/j.jbc.2024.107695 (PMC11416245; doi:10.1016/j.jbc.2024.107695)
Supplement: Supplemental information [file mmc1.pdf]

SUPPLEMENTAL INFORMATION

Cell cycle arrest combined with CDK1 inhibition suppresses genome-wide mutations by activating alternative DNA repair genes during genome editing.

Nozomi Fukuda, Keisuke Soga, Chie Taguchi, Jumpei Narushima, Kozue Sakata1, Reiko Kato, Satoko Yoshiba, Norihito Shibata, Kazunari Kondo

Supplementary Figures

Fig.S1. Characteristics of TSCE 5, TSCE2 and TSCE122 cells, and patterns of DNA breaks induced by gRNA3/Cas9 at the target site.

Fig.S2. Cell cycle synchronization of TSCE5 cells. Double thymidine block (d-thy) and colcemid treatments were used to arrest cell cycle at S–G2/M phase.

Fig.S3. Comparison of indels and base substitutions induced by I-*Sce*I editing in TSCE5 cells under non-syn and S~G2/M conditions.

Fig.S4. Naturally occurring mutations in resting cells.

Fig.S5. Doublet base substitution signatures (DBS). DBS of cells from (*A* and *B*) large and (C) small colonies were analyzed under non-syn and S–G2/M conditions.

Fig.S6. Copy number alterations (CNA) of all chromosomes in genome-edited TSCE5 cells.

Supplementary Tables

Table S1. Table.S1. SNVs and indels in TSCE5cells edited with gRNA3/Cas9 (A) or I-SceI (B) under non-syn conditions

Table S2. Table.S2. SNVs and indels in TSCE5cells edited with Cas9/gRNA-3 or I-SceI under S~G2/M conditions

Table S3. Table S3. Structural variants in genome-edited TSCE5 cells under non-syn and S~G2/M conditions.

Table S4. Table S4. gene expression profiles in gRNA3/Cas9-edited cells under S-G2/M conditions compared to unedited control cells.

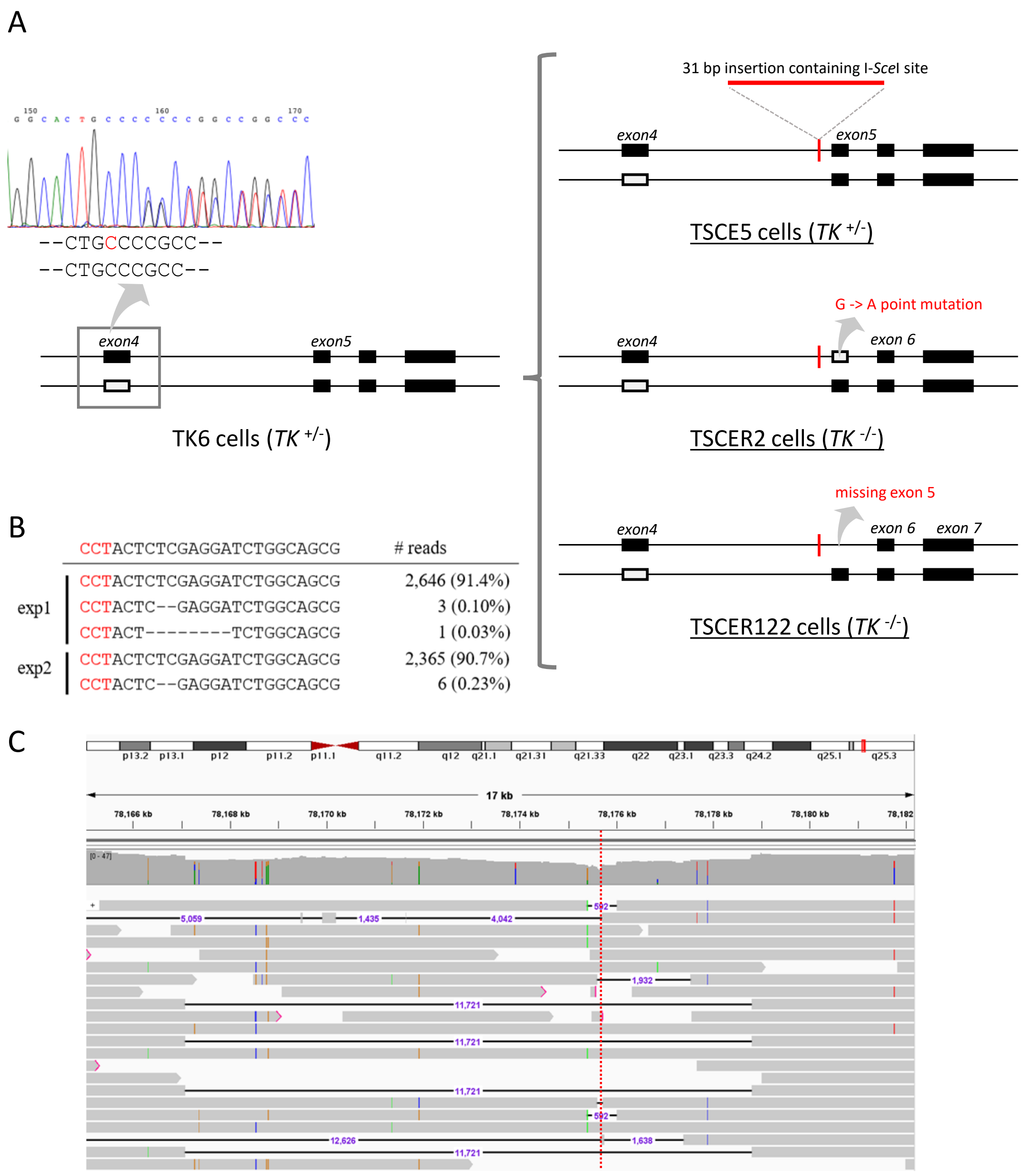

Figure S1. Characteristics of TSCE 5, TSCE2 and TSCE122 cells, and patterns of DNA breaks induced by gRNA3/Cas9 at the target site.

A, Thymidine kinase (TK) assay was used to select TSCE5 cells with deletions > 81 bp using trifluorothymidine (TFT). Similarly, TK assay selected cells repaired by homologous recombination (HR) using deoxycytidine and hypoxanthine-aminopterin-thymidine (CHAT) from TSCER2 and TSCER122 cell lines. TSCER2 carries 1 bp mutation in exon 5, whereas TSCER122 lacks 335 bp, including entire exon 5.

B, gRNA3/Cas9 editing induced two or eight bp deletions at low frequencies (0.03 to 0.23%) in TSCE5 cells at target site described in Fig. 1B.

C, Larger deletions were detected in gRNA3/Cas9-edited TSCE5 cells at target site. Sequence was analyzed using Nanopore Long-Read Sequencer. Red dotted line indicates target site of gRNA3. Larger deletions ranged from 592 to > 10,000 bp.

asynchronous (*non-syn*)

Control			
cell cycle	Average		SD
late G2/M	6.5	±	3.97
S	32.3	±	12.07
G1/G0	61.2	±	15.94
(n = 6)			

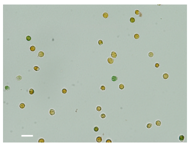

synchronized (*S~G2/M*)

doule Thy - Colcemid			
cell cycle	Average		SD
late G2/M	20.0	±	6.76
S	44.3	±	4.42
G1/G0	35.7	±	5.59
(*n* = 33)			

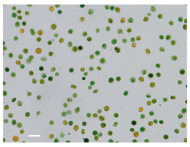

synchronized (*d-thy alone*)

doule Thy			
**cell cycle**	**Average**		**SD**
late G2/M	5.6	±	2.39
S	32.6	±	4.07
G1/G0	61.7	±	5.4
(*n* = 10)			

Figure S2. Cell cycle synchronization of TSCE5 cells. Double thymidine block (d-thy) and colcemid treatments were used to arrest cell cycle at S–G2/M phase. Treatment induced 64.3% S–G2/M synchronization. Synchronization was visualized using cell cycle assay Cell-Clock reagent. Cells in yellow or green indicate G1 or S phase, and cells in deep green to dark blue indicate G2/M phase. Furthermore, 6 to 33 independent experiments were performed (*n* = 6 or 33). Then cyclin dependent kinase (CDK) inhibitor Ro3306 was added to synchronized cells at genome editing. Scale bar, 25 μm.

Numbers of genome-wide mutations generated during I-*Sce*I editing

A

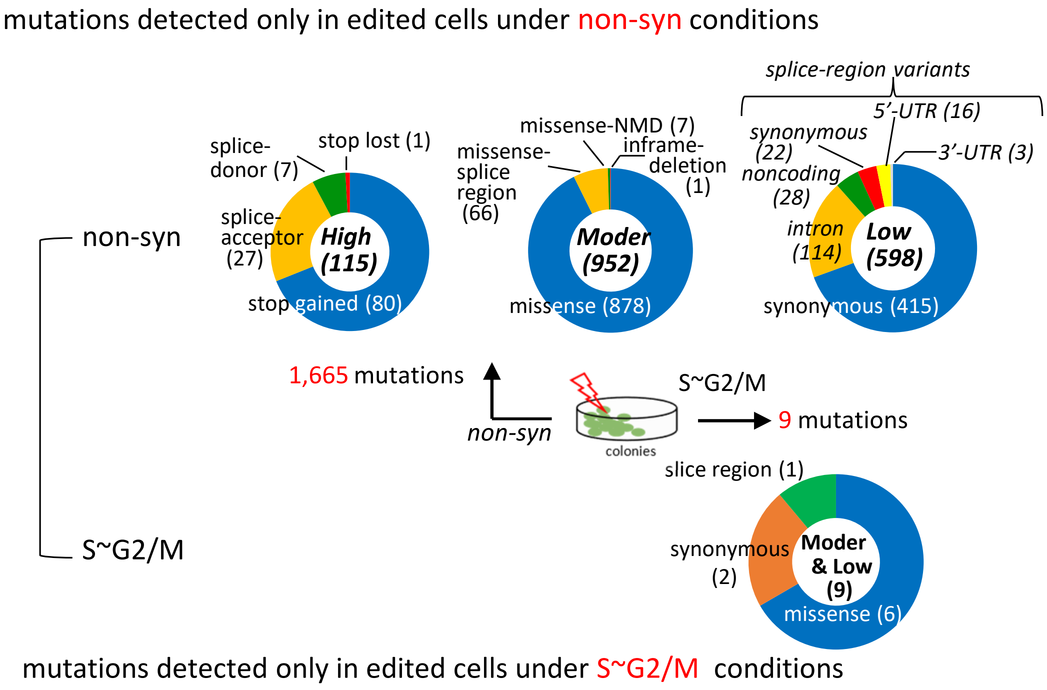

B

**Common genes unintentionally edited by both gRNA3/Cas9 and I-*Sce* I editing under non-syn conditions**					
ABCA6	CCHCR1	GOLGA3	LAMA3	PSD2	TNN
AC011473.4	CDH8	GPAM	LMLN2	PTK7	TP53BP1
AC106741.1	CEP290	GPR179	LRP1	QSER1	TRPM1
ADAMTS14	CFTR	GPR84	MAP1A	RBM15B	TTC19
ADCY9	CIITA	GRIK5	MFSD5	RNF220	TTN
ADGRF5	CMYA5	GRM7	MRC1	SCARB2	UBE3C
AGL	CNTNAP4	HLA-DRB6	MT-CO1	SCN3A	UNC13B
ANKRD30B	COL10A1	HRNR	MTMR12	SCN5A	UNC13D
APC	COL7A1	IFFO1	MUC4	SIPA1L2	UNC79
ARMC9	CRYBG3	IFT122	MUC5AC	SNCAIP	VPS50
ATM	CYLD	IQGAP3	MYH7	SNX5	WDFY3
BBS7	DCC	KALRN	OR2T2	SPTBN4	ZNF169
BORA	DNAH10	KCNK2	OR2T34	ST6GALNAC2	ZNF462
BSN	EDAR	KCNQ1	OTOGL	SVIL	
C12orf42	ELK4	KIAA2026	PDE10A	SYNE1	
C2CD5	EVPL	KLK9	PIGN	TAF5	
C2orf16	F11	KMT2C	PKD1	TAPT1	
C3orf49	FAM221B	KNTC1	PKHD1L1	TCHH	
CARD11	FMN2	KRT35	PLXNB1	TEX15	
CASR	FRAS1	LAMA1	PRPF40A	TLN2	

Figure S3. Comparison of indels and base substitutions induced by I-*Sce*I editing in TSCE5 cells under non-syn and S~G2/M conditions. Genome-wide mutations were extracted by comparing non-edited control cells with I-*Sce*I-edited cells, using the Mutect2 software in GATK. ***A,*** I-*Sce*I-edited cells generated 1,665 and 9 mutations in non-syn and S~G2/M conditions, respectively. Like gRNA3/Cas9-edited cells, the number of mutations was suppressed under S~G2/M conditions. Six missense, 2 synonymous and one splice-region mutation was observed. ***B,*** Common genes detected in both gRNA3/Cas9- and I-*Sce*I-edited cells under non-syn conditions.

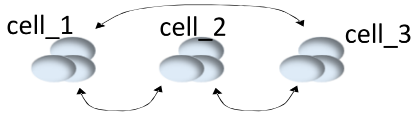

TSCE5

cell_1 vs 2
MUC5AC
LUZP2
PARP4
PWRN1
ATP10A
COL1A1
LRRTM4
COL4A3
RRBP1
FRG1EP
CCR9
PCDHB11
ADGRF1
EIF3B
SERPINA7
SLC25A5

RPE-1

cell_1 vs 2	cell_2 vs 3	cell_3 vs 1
ACAN	ABCC12	ACSL6
ADPRHL1	AGAP9	ANKRD20A21P
AKAP9	CABIN1	ANKRD36C
ALG1L9P	CSPG4	AOAH
ASPN	DNAH10	ARHGAP25
CCNYL1	EMD	ATP6V1B2
CDV3	F5	CHAF1A
CEP131	GOLGA6L2	DNAH14
FGF19	HCN2	ENOSF1
FRG1DP	KIR2DL4	FAM230F
GDPD3	KLRC2	FCGBP
HEXD	KRTAP4-7	FLG2
HLA-B	LARGE2	FOXD4L1
IMPA1P1	MUC4	HCN1
KIF4CP	MXRA5	IGHV4-4
KIR3DL1	NGEF	KIR2DL4
LAIR1	OR2L8	KLHDC3
LMAN2	PNPLA6	MUC3A
MSRB3	PROB1	NAIF1
MUC17	PRSS1	PKN3
MUC22	SERPINA1	PWP1
MUC3A	SETD7	SHANK2
MUC4	SKA3	SLAMF7
MUC5B	SLC28A2	SLC25A3
NBEAL1	TAOK1	SNX27
NUDT17	TMEFF1	SPDYE1
OR8U1	VAPA	STK24
PWWP4	WASH4P	SYPL1
RASGEF1A	WDR26	TEX13D
SDR42E2		TMEM169
SLC24A1		TMTC2
TAFA5		TRIOBP
TFDP1		TXNDC15
UGGT2		ZDHHC11
WDR89		ZFPM2
ZNF71		
ZNF750		
ZSWIM6		

Figure S4. Naturally occurring mutations in resting cells.

Frequencies of naturally occurring mutations were analyzed using next-generation sequencing (NGS) in two or three groups of untreated retinal pigment epithelial (RPE)-1 cells. Two- or three-cell groups were cultured separately for days to weeks, and then genomic DNA (gDNA) was extracted. Somatic mutations in the three cell groups were investigated using GATK/Mutect2. Results showed 16 variants in TSCE5 cells, and 29, 35, and 38 in the three groups of untreated RPE-1 cells. Different genes were mutated among the groups, although some such as *REP-1*; mucin 3A, cell surface associated (*MUC3A*); and killer cell immunoglobulin like receptor, two Ig domains and long cytoplasmic tail 4 (*KIR2D4*) were common.

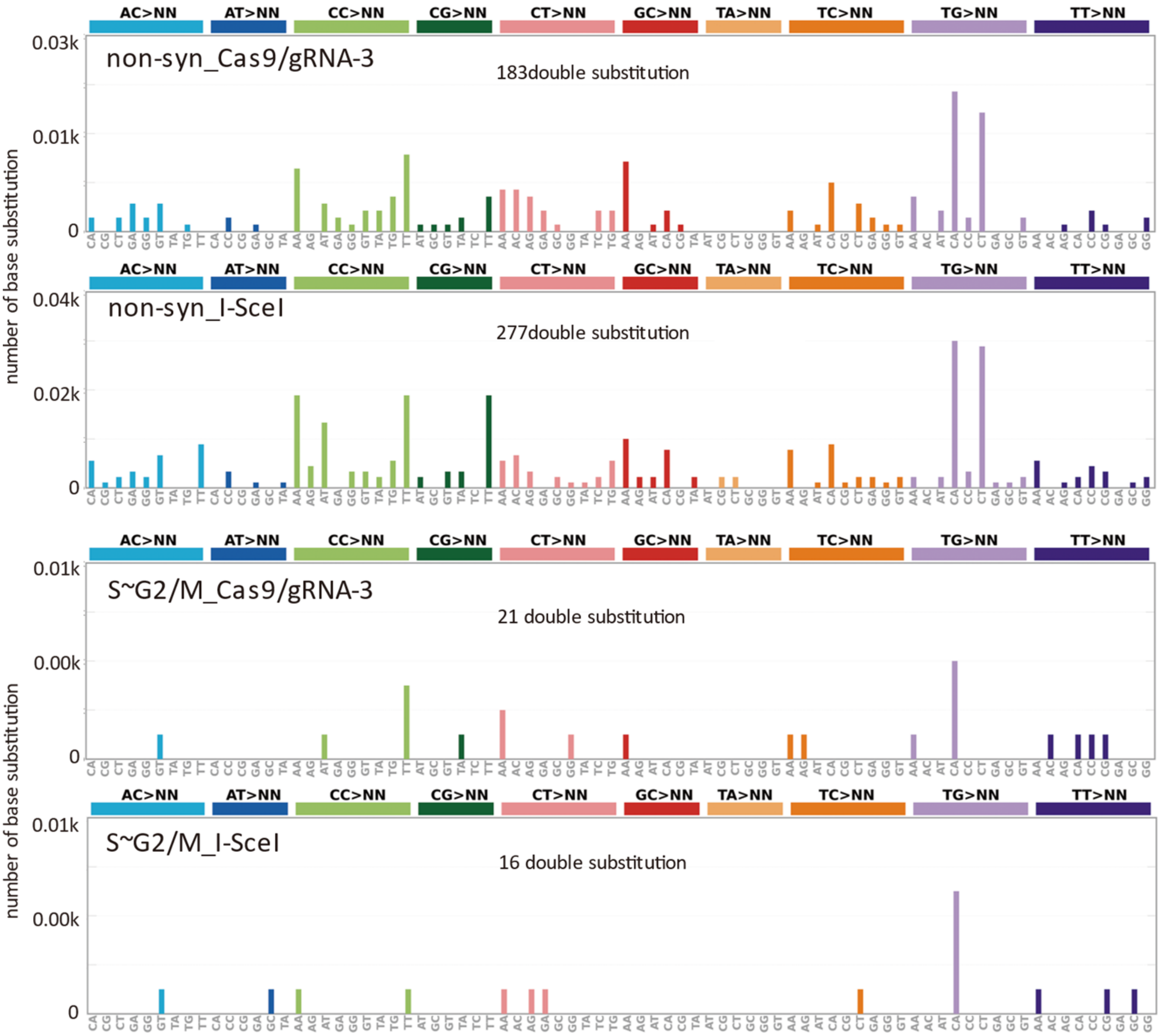

Figure S5. Doublet base substitution signatures (DBS).
DBS of cells from (*A* and *B*) large and (C) small colonies were analyzed under non-syn and S–G2/M conditions. Cells had fewer substitutions under S–G2/M conditions. Base substitution patterns were similar between non-syn and S–G2/M conditions. Number of substitutions in synchronized cells was one-tenth lower than that in cells under non-syn conditions.

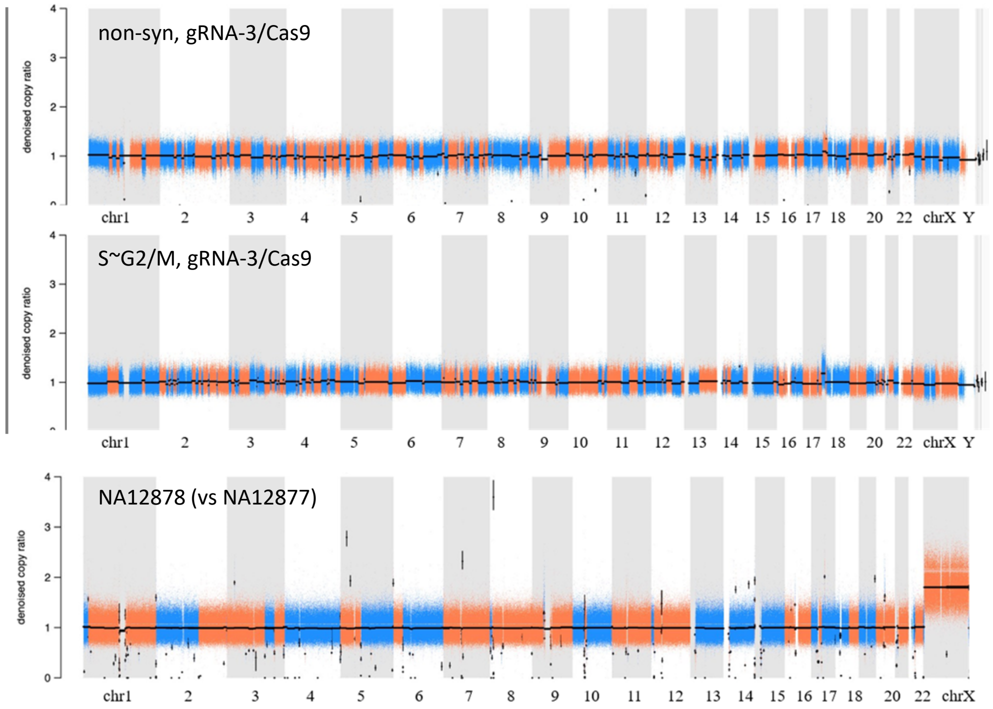

Figure S6. Copy number alterations (CNA) of all chromosomes in genome-edited TSCE5 cells. Guide RNA (gRNA3)/clustered regularly interspaced short palindromic repeats (CRISPR) associated protein 9 (Cas9)- and I-*Sce*I-edited cells under non synchronous (non-syn) and S–G2/M arrest followed by cyclin dependent kinase 1 (CDK1) inhibition (S–G2/M) conditions were used for CNA analysis. NA12877 and NA12878 were used as positive quality controls to demonstrate precision of ModelSegments in GATK (*bottom*). Denoised copy ratios (Y-axis) are indicated as relative ratios of control; therefore, ratio of 1 indicated no CNA.
